# Ophthalmic artery occlusion following neuro-embolization of the external carotid artery, a case report

**DOI:** 10.1186/s12886-017-0490-7

**Published:** 2017-06-15

**Authors:** Ng Wei Loon, Balwant Singh Gendeh, Rozman Zakaria, Jemaima Che Hamzah, Norshamsiah Md Din

**Affiliations:** 1 Department of Ophthalmology, Universiti Kebangsaan Malaysia Medical Center, Jalan Yaacob Latiff, 56000 Kuala Lumpur, Malaysia; 2 Department of Otorhinolaryngology, Universiti Kebangsaan Malaysia Medical Center, Jalan Yaacob Latiff, 56000 Kuala Lumpur, Malaysia; 3 Department of Radiology, Universiti Kebangsaan Malaysia Medical Center, Jalan Yaacob Latiff, 56000 Kuala Lumpur, Malaysia

**Keywords:** Ophthalmic artery occlusion, Neuro-embolization, Microspheres, Case report

## Abstract

**Background:**

Embozene® is a new neuroembolizing microsphere used to reduce intraoperative bleeding for head and neck tumours. We report a case of iatrogenic ophthalmic artery occlusion after Embozene® embolization of the external carotid artery (ECA).

**Case presentation:**

A 22-year-old African gentleman presented with left nasal obstruction and epistaxis for 2 years and was diagnosed with nasopharyngeal carcinoma. He subsequently underwent embolization of the maxillary branch of the left ECA using Embozene® Microspheres - 250 μm in size before endoscopic tumour excision to reduce intra-operative bleeding. He complained of sudden painless profound visual loss in the left eye (LE) two hours after embolization. Visual acuity in LE was no light perception. Fundus examination showed pale retina with no cherry red spot. Arterial narrowing and segmentation were seen in all quadrants. A diagnosis of left ophthalmic artery occlusion was made. Despite immediate management including ocular massage and lowering of intraocular pressure, the visual loss remained. Retrospective review of digital subtraction angiogram showed an anastomosis between the left ophthalmic artery and anterior deep temporal artery as a potential route for microspheres migration.

**Conclusion:**

Pre-operative angio-architecture understanding and diligent selection of embolic material are helpful in preventing this adverse event. The use of newer agents for embolotherapy may cause migration of embolic material from the external to the internal carotid system leading to ophthalmic artery occlusion and blindness.

## Background

Embolization of extracranial tumours in the head and neck region has been used to facilitate the treatment of intractable epistaxis or hypervascular tumors. Commonly used particles include gelatine sponge, polyvinyl alcohol (PVA), trys-acril gelatine microspheres or coils. The complication rate varies between2.2% to 25%. [1, 2] Cranial nerve palsies, stroke and blindness are among those reported [3, 4].

Embozene® Microspheres (CeloNova BioSciences Inc.) is a new microsphere used in neuroembolization. It is made from smooth and deformable hydrogel core (polymethylmethacrylate) coated with a shell (Polyzene ®-F) [5] which ovalizes when confined, allowing deeper penetration. It comes in different sizes varying from 40 μm to 1300 μm. We report a case of blindness due to the inadvertent migration of these spheres between the external carotid artery (ECA) and internal carotid artery (ICA) system.

## Case presentation

A 22-year-old African gentleman presented with left nasal obstruction and epistaxis for 2 years. Examination revealed a nasopharyngeal mass which upon biopsy revealed an undifferentiated nasopharyngeal carcinoma stage III. He had radiotherapy but in view of persistent symptoms and poor response to radiotherapy, endoscopic tumour excision was planned. Prior to that, he underwent embolotherapy of the maxillary branch of the left ECA under sedation and local anaesthesia. The indication for embolization was intractable epistaxis and to reduce intraoperative bleeding during tumour excision. Intermittent Embozene® Microspheres 250 μm injection under fluoroscopy guidance was done before deployment of a 2 mm × 5 mm coil. He was not given heparin or anticoagulant during or after the procedure.

**Fig. 1:**
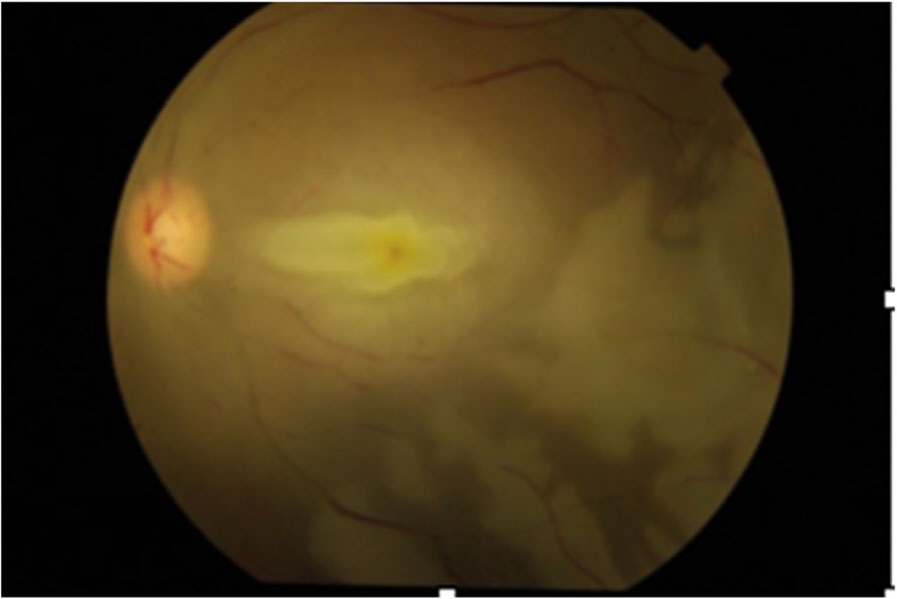
The appearance of the fundus 24 h after the onset of sudden visual loss. The neurosensory macula was thickened and raised. The retinal arteries were diffusedly narrowed and segmented. Areas of retinal pallor were better defined

Approximately 2 h after the procedure, the patient complained of sudden painless profound visual loss in the left eye (LE). Visual acuity in the LE was no light perception with a dense afferent pupillary defect. Funduscopy examination showed a pale retina without cherry red spot; and generalized arterial attenuation (Fig. 1). The other eye was completely normal. He did not have neurological deficit.

**Fig. 2:**
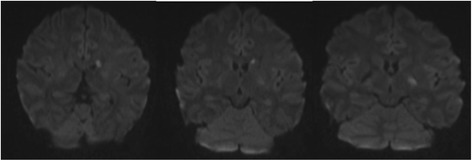
MRI images showing hyperintense lesions involving the left caudate nucleus and basal ganglia on DWI sequence due to restricted diffusion

**Fig. 3:**
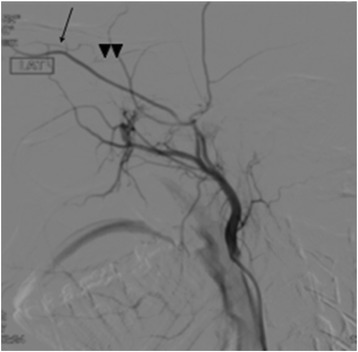
Cerebral angiogram. Arrow shows the communication between deep anterior temporal artery (branches of ECA) and lacrimal artery (branches of ICA, *arrow*). Note the faint retrograde opacification of the ophthalmic artery (*arrow heads*)

A diagnosis of left central retinal artery occlusion was made but the absence of a cherry red spot further suggests the absence of choroidal circulation indicating a possible occlusion of the ophthalmic artery as well. Ocular massage and immediate lowering of intraocular pressure with intravenous acetazolamide 500 mg stat and topical antiglaucomas failed to restore the retinal circulation, with visual acuity remaining no light perception after 24 h. We did not do AC paracentesis as the patient was drowsy from the sedation. MRI of the brain and orbit showed high signal intensity foci on diffusion weighted image (DWI) involving the left caudate nucleus and basal ganglia (Fig. 2), suggestive of small infarctions. The patent however did not show any clinically apparent neuro-deficits from these infarcts. Cerebral angiogram revealed collaterals between branches of the ECA and lacrimal artery (a branch of the internal carotid artery (ICA) (Fig. 3). The patient however declined fundus fluoresecence angiography and other ocular investigations. His vision remained no light perception on the last follow-up three months later.

## Discussion

Embolization of head and neck tumours facilitates surgery by reducing intra-operative bleeding thus allowing better visualization. However, reflux or passage of embolic materials through the ECA-ICA anastomoses has been responsible for devastating complications [1–4].

Because blood flow to the brain and eye is predominantly from the ICA system, embolization of the ECA was thought to be relatively safe. This case proves the contrary as the following internal maxillary artery collaterals may result in inadvertent embolic agent migration: recurrent meningeal artery - orbital branches of middle meningeal artery, anterior deep temporal artery - lacrimal artery, and sphenopalatine artery/distal internal maxillary artery - ethmoidal arteries. [6] Thorough understanding of the vascular supply, hemodynamic characteristics, venous outflow patterns, and collaterals is therefore paramount for safe and effective treatment. Prior identification of all possible anastomoses is important before the injection of such agents.

PVA is a commonly used material. It is irregular and therefore causes clumping and clogging of microcatheters or large vessels. Smaller particles (<250 μm) readily cross ECA–ICA anastomoses. Particles more than 250 μm in size are generally too large to cross anastomoses and hence preferred in head and neck tumors. [7] Simultaneous central retinal artery (CRA) and ciliary artery occlusion after embolization of the left internal maxillary artery (IMA) with PVA (200 μm in size) has been reported, postulating migration of particles via the lacrimal artery into the ICA system. Visualization of a choroidal blush during ECA angiography indicating collaterals to the eye was identified as an indicator for the presence of the ECA-ICA anastomoses. [4] There has also been other report on ophthalmic artery occlusion following facial filler injections [8].

Embozene® is a new embolizing microsphere. The smooth surface allows it to travel into deep structures of a tumour. This newer class microembolic agent is soft, deformable and tends to ovalize when confined, a trait that makes this agent more effective. Although traditional PVA continue to have their places in embolotheraphy, the market is shifting to spherical particle. Nonetheless, smoother and smaller microspheres tend to pass through the anastomoses more readily. Some authors generally do not recommend the use of particles smaller than 150 μm to avoid potential embolic complication. Embozene 250 μm in size was used in this patient. Although the size used is larger than previously used materials, it could still migrate into the ICA circulation, possibly because of its unique features. Performing the procedure under sedation and local anaesthesis may also contribute to this devastating complication. The patient may not be completely relaxed under local anesthesia and any inadequate pain control may induce vasospasm [9] resulting in inadequate visualization of existing collaterals. Abnormal blood supply via branches of the internal carotid artery especially for midline tumours has also been reported and this possibility cannot be ruled out [10, 11].

We postulated that simultaneous CRA and ciliary artery occlusion occurred in this patient as there was no cherry red spot to indicate choroidal circulation. The microspheres might have refluxed into the ophthalmic artery and subsequently the ICA via the deep anterior temporal artery (branches of IMA) and lacrimal artery (branches of the ophthalmic artery). The use of newer agents such as Embozene, although with excellent penetration characteristics, need a higher cut-off size to prevent the migration through existing anastomoses as generally believed.

## Conclusion

Understanding the pre-operative angio-architecture and diligent selection of embolic material are helpful in preventing this adverse event. We recommend embolization to be performed under general anaesthesia to allow complete relaxation and thorough visualization of existing collaterals. Larger and irregular embolic materials should also be considered.

## Abbreviations

CRA: Central retinal artery
DWI: Diffusion weighted image
ECA: External carotid artery
ICA: Internal carotid artery
IMA: Internal maxillary artery
LE: Left eye
MRI: Magnetic resonance imaging
PVA: Polyvinyl alcohol
